# Glycosylated VCAM-1 isoforms revealed in 2D western blots of HUVECs treated with tumoral soluble factors of breast cancer cells

**DOI:** 10.1186/1472-6769-9-7

**Published:** 2009-11-22

**Authors:** Delina Montes-Sánchez, Jose Luis Ventura, Irma Mitre, Susana Frías, Layla Michán, Aurora Espejel-Nuñez, Felipe Vadillo-Ortega, Alejandro Zentella

**Affiliations:** 1 Departamento de Medicina Genómica y Toxicología Ambiental, Instituto de Investigaciones Biomédicas, UNAM. Ciudad Universitaria, Circuito Interior apartado postal 70228, CP04510, México DF; 2 Departamento de Bioquímica, Instituto Nacional de Ciencias Médicas y Nutrición "Salvador Zubirán", SSA Vasco de Quiroga no 15 Colonia Sección XVI, Delegación Tlalpan. CP.4080, México DF; 3 Dirección de Investigación Básica, Instituto Nacional de Cancerología, SSA Av San Fernando no 22 Col. Sección XVI, Tlalpan, CP 14080, México DF; 4 Dirección de Investigación, Instituto Nacional de Perinatología, SSA Montes Urales 800 Torre de Investigación 5° piso, Colonia Lomas Virreyes, Delegación Miguel Hidalgo, CP11000, México DF; 5 Edificio de Investigación, Facultad de Medicina, UNAM. Ciudad Universitaria, Circuito Interior apartado postal 70228, CP04510, México DF

## Abstract

**Background:**

Several common aspects of endothelial phenotype, such as the expression of cell adhesion molecules, are shared between metastasis and inflammation. Here, we analyzed VCAM-1 variants as biological markers of these two types of endothelial cell activation. With the combination of 2-DE and western blot techniques and the aid of tunicamycin, we analyzed N-glycosylation variants of VCAM-1 in primary human endothelial cells stimulated with either TNF or tumoral soluble factors (TSF's) derived from the human breast cancer cell line ZR75.30.

**Results:**

Treatments induced a pro-adhesive endothelial phenotype. 2D western blots analysis of cells subjected to both treatments revealed the expression of the two known VCAM-1 isoforms and of previously unknown isoforms. In particular TSFZR75.30 induced an isoform with a relative molecular mass (Mr) and isoelectric point (p*I*) of 75-77 kDa and 5.0, respectively.

**Conclusion:**

The unknown isoforms of VCAM-1 that were found to be overexpressed after treatment with TSF's compared with TNF, could serve as biomarkers to discriminate between inflammation and metastasis. 2D western blots revealed three new VCAM-1 isoforms expressed in primary human endothelial cells in response to TSF stimulation. Each of these isoforms varies in Mr and pI and could be the result of differential glycosylation states.

## Background

Endothelial cells line the inside of all blood vessels forming an interface between circulating blood and the underlying tissues. As such, endothelial cells comprise, a critical metabolic organ involved in the generation and regulation of multiple physiological and pathological processes such as coagulation, hemostasis of local vascular pressure, inflammation, atherosclerosis, angiogenesis, and metastasis [1]. In the context of the current model of tumoral dissemination, glycoproteins including cell adhesion molecules are expressed on the apical endothelial membrane, interacting with counter-receptors on circulating cancer cells, facilitating the spread of the disease [2,3].

Glycoproteins are prominent constituents of biological membranes, and are involved in various biological functions, including immunological protection, enzymatic catalysis, hormonal control, ion transport, structural support, molecular recognition, and cell adhesion. Structurally, glycoproteins are comprised of a peptide backbone, with carbohydrate chains covalently attached to asparagine (*N*-glycan) or serine/threonine (*O*-glycan) residues [4,5]. The immunoglobulin super family of cell adhesion molecules (IgCAMs) constitutes a large group of cell surface glycoproteins that are specialized for cell-cell adhesion [6,7]. Recently, these molecules have been reported to play an important role in pathological processes, including tumor invasion and metastasis [8,9]. In particular, vascular adhesion molecule-1 (VCAM-1) is a cell surface glycoprotein expressed on the apical membrane of endothelial cells activated by cytokines [10,11]. Two isoforms of VCAM-1 have been reported, a full length protein (a) and a smaller version (b) lacking exon 5. Their expression occurs in response to inflammatory mediators and is dependent on the translocation of the transcription factor NF-κB into the nucleus [12]. VCAM-1 interacts with its integrin counter receptor very late antigen-4 (VLA-4), to mediate the recruitment of leukocytes [13-15]. The adhesion of tumor cells to the apical endothelial membrane resembles their interaction with leukocytes when endothelial cells have been activated with tumor necrosis factor (TNF). TNF is a pro-inflammatory cytokine, principally derived from mononuclear phagocytes, that induces transient phenotypic changes in the endothelial cells, transforming their apical membrane from a quiescent non-adhesive to an activated pro-adhesive surface amenable for cell-cell interactions [16-18].

We have previously reported that the tumoral soluble factors (TSF's) with a very low to none TNF content can induce a strong pro-adhesive phenotype similar to the one induced by TNF [19-21]. Here we analyzed changes in the content of VCAM-1 in endothelial cells under experimental treatments with TNF or TFS's that led to an increase in tumor cell adhesion. We combined enhanced chemi-luminescent sensitive (ECL) detection with the high resolving power of two-dimensional polyacrylamide gel electrophoresis (2-DE), using these techniques and the aid of tunicamycin were able to identify undescribed isoforms of VCAM-1.

## Results

### Tumoral soluble factors (TSF's) secreted by human breast cancer cell lines induced adhesion of U937 myelomae cells to HUVECs

**Figure 1:**
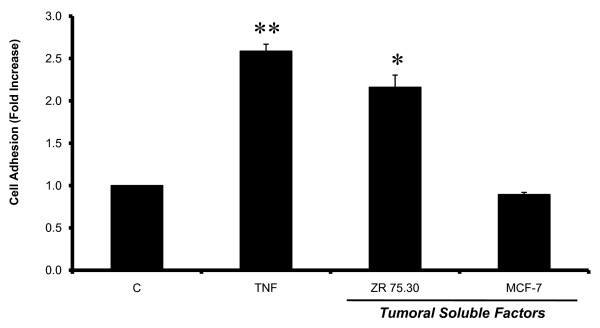
**Tumoral soluble factors derived from breast cancer cell line ZR75.30 increase adhesion**. The cell adhesion assay shows the endothelial activation, which is proportional to the adhesion of human myeloma U937 cells to HUVECs. Each bar represents the average of three independent experiments performed in triplicates. Statistical significance when compared to the control group is defined as: p < 0.05 (*) and p < 0.01 (**). The error bars indicate ± standard error of the mean.

Using an *in vitro *cell adhesion assay, we found that tumor soluble factors (TSF's) induced an activated phenotype of HUVECs (Figure 1). The cells were stimulated for 3 h with either TNF (10 ng/ml) or TSF's derived from breast cancer cells lines: MCF-7 and ZR75.30 (1 μg/ml), (labeled TSFMCF-7 and TSFZR75.30, respectively), diluted in M-199 medium. Then, the medium containing TNF or TSF's was removed, and the HUVECs were coincubated for 3 more h with previously radio-labeled U937 cells as was described in methods. Compared to untreated control cells, HUVECs treated with TNF presented a 2.6 fold increase in cell adhesion, while those treated with TSFZR75.30 had a 1.5 induction. The soluble factors secreted by the other breast cancer cell line (TSFMCF-7) did not show any adhesive effects. These results indicate that TSFZR75.30 induced a pro-adhesive phenotype similar to that induced by TNF.

### Biochemical contents of TSFZR75.30

We used a Bio-Plex system™ by BIO-RAD, CA. [16,22] to evaluate the content of cytokines, chemokines, and growth factors in TSFZR75.30. The results are summarized in Table 1. TSFZR75.30 contained four cytokines (IL-6, IL-8, G-CSF, MCP-1) that were present at levels above the maximum measured limit. Nine cytokines (IL-2, IL-4, IL-10, IL-5, IL-7, IL-9, IP-10, MIP-1α, RANTES) were below the minimum measurable limit, and thirteen were present at low levels within the detectable range, TNF was present in this group. In comparison with the amount used (10 ng/ml), TNF was 50 times less abundant (0.22 ng/ml) in the mixture of TSFZR75.30. Taken together, these results suggest that TSFZR75.30 contain cytokines, chemokines, and growth factors that are able to induce a proadhesive phenotype in endothelial cells.

**Table 1:** Elements contained (pg/ml) in TSFZR75.30 examined by Bio-Plex array.

	*TSFZR75.30*	*Maximum detected*	*Minimum detected*
IL-2	<1.3	21517	1.3
IL-4	<0.2	3854	0.2
IL-6	**>33053**	33053	2.0
IL-8	**>24800**	24800	1.5
IL-10	<1.9	30108	1.9
GM-CSF	354	10791	0.6
IFNγ	112.5	40543	2.5
TNF	22.5	70463	4.3
IL-1β	49.9	37082	2.3
IL-5	<2.4	39452	2.4
IL-7	<2.7	44339	2.7
IL-12	12.5	41843	2.5
IL-13	29.5	33114	2.0
IL-17	13	26740	1.6
G-CSF	**>28728**	28728	1.7
MCP-1	**>27978**	27978	1.7
MIP-1β	15.5	37041	2.2
IL-1rα	130.5	42658	2.6
IL-9	<1.6	27064	1.6
IL-15	13	26368	1.6
Eotaxin	8.5	24294	1.5
FGF-basic	9	18355	1.1
IP-10	<4.3	69784	4.3
MIP-1α	<1.1	18493	1.1
PDGF-BB	14.5	32062	1.9
RANTES	<1.5	24678	1.5
VEGF	**11590.5**	39592	2.4

### TSFZR75.30 induced the expression of VCAM-1 isoforms in HUVECs that disappeared when the cells were pre-treated with tunicamycin

**Figure 2:**
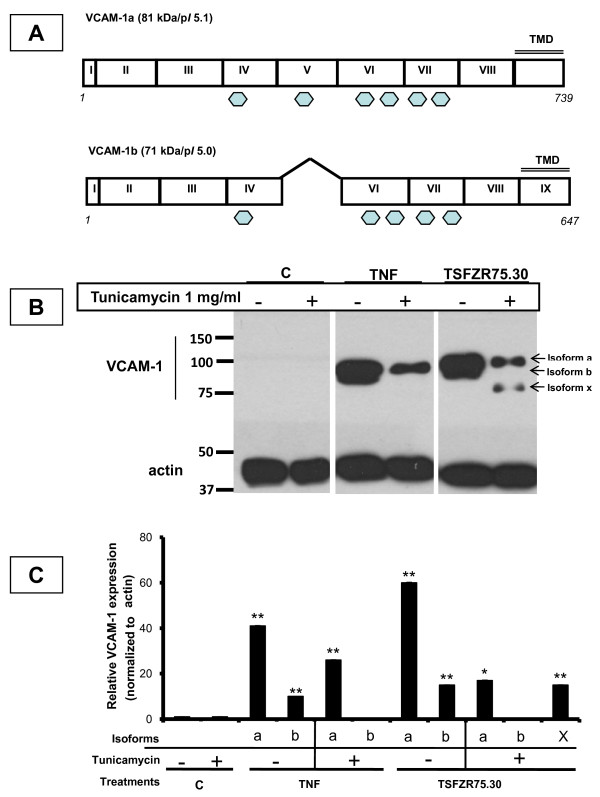
**Expression of VCAM-1 cell content is increased when HUVECs are treated with TSFZR75.30 and decreased when cells are pre-treated with tunicamycin**. A) Exon maps of VCAM-1a: 739 amino acid, 9 exons and 6 N-glycosylation sites and, VCAM-1b: 647 residues, lacking exon 5 and the 2nd N-glycosylation site (hexagons). B) Western blot to detect VCAM-1 in twenty micrograms of total extracts from HUVECs treated for 3 hr with TNF or TSFZR75.30 (lanes 3,5) or pre-treated with tunicamycin 3 h before TNF or TSF's (lanes 4,6). C) Histogram of the mean of three individual experiments and the normalized densitometric values reflecting expression of the VCAM-1 isoforms: a, b and x with respect to actin signal. p < 0.001 (*) compared control. The error bars indicate ± standard error of the mean.

VCAM-1 is a classic cell adhesion molecule, with a Mr ~80-110, that is expressed during the inflammatory response and has also been associated with tumoral dissemination [23-25]. VCAM-1 is a surface molecule reported to have two isoforms: i) the complete form (a), composed of 739 amino acid residues encoded by nine exons and containing six N-glycosylation sites that are indispensable for protein function during molecular recognition with integrins, and ii) the small form (b), which is composed of 647 residues and results from an alternative splicing event that removes exon 5, eliminating the second N-glycosylation site (Figure 2A). The changes in the cellular content of VCAM-1 were evaluated by western blots analysis from total extracts of HUVECs treated with either TNF or TSFZR75.30 for 6 h (Figure 2B). The content of VCAM-1 in cells treated with TNF (10 ng/ml) and TSFZR75.30 (1 μg/ml) was increased. The two reported isoforms appeared as a tight doublet (VCAM-1a, Mr ~90-95 kDa and VCAM-1b ~80-83 kDa) (lanes 3 and 5). Compared to the control, VCAM-1a isoform increased 41 and 60 fold, while isoform VCAM1b increased 10 and 15 fold. However, when we pre-treated cells for 3 h with tunicamycin (1 μg/ml), an inhibitor of N-glycosylation, only the band corresponding to isoform "a" was visible in TNF-treated HUVECs (lane 4). In the case of treatment with the TSFZR75.30, a new isoform was revealed, with a Mr ~77 kDa, in addition to isoform "a" (lane 6). In both cases isoform "b" was absent. Expression of isoform "a" and the new isoform, labeled as "x", was increased 17 and 15 fold respectively, to the control. It is clear that tunicamycin had a differential effect on the glycosylation state and the content level of VCAM-1 isoforms, leading to the disappearance of isoform "b" and the emergence of a undescribed VCAM-1 isoform in cells treated with TSFZR75.30, labeled here as isoform "x". When we performed a cell adhesion assay similar to the one describe in Figure 1, on cell that had been pretreated with tunicamycin (1 μg/ml) for 3 h. The fold increase in cell adhesion induced by TSFZR75.30 fell from 3.1 ± 0.5 to 1.7 ± 0.3 in control HUVECs compared to cells pretreated with tunicamycin. These results indicate that 50% of the observed cell adhesion is dependent from N-glycosylations.

### 2-DE analysis of total extracts of HUVECs treated with TNF or TSFZR75.30

**Figure 3:**
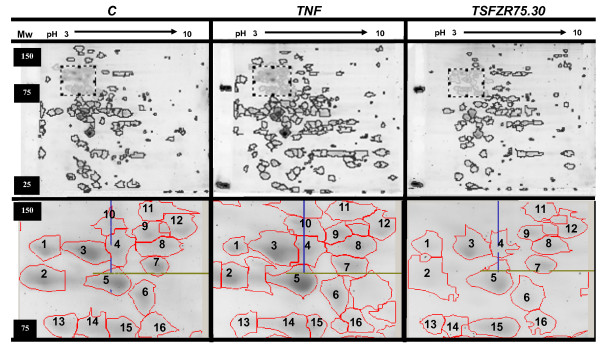
**Informatic analyses with the program Image Master 2D Platinum™**. 50 μg of total extracts were loaded and stained with the Deep-Purple fluorochrome™. The 1^st ^dimension was resolved in a linear pH-range from 3-10 and in 10% acrylamide for the 2^nd ^dimension. Control (C): left panels; TNF: central panels; TSFZR75.30: right panels. Lower panels represent an amplified view of the dotted rectangle in the upper panels. Patterns are the representative images of three individual experiments each performed in duplicate.

In an attempt to further characterize the different VCAM-1 isoforms visualized in Figure 2B, we prepared 2-DE gels that were stained with a fluorescent dye (Deep purple™) and analyzed, focusing on the regions expected to contain these isoforms. Two dimensional electrophoresis of total extracts from HUVECs treated with TNF presented 191 spots, 24% more than control cells (154 spots) or those treated with TSFZR75.30 (150 spots) (Figure 3, upper panels). The dotted rectangles correspond to the position (Mr and p*I*) predicted for the VCAM-1 isoforms. An amplified view of this region appears in the lower panels; 16 spots were detected with variations in intensity between treatments. However, none of the spots replicated the increase in the amounts of VCAM-1 isoforms observed in the westerns blots from Figure 2B. It is possible that the amount of protein on the gels was not enough to visualize the VCAM-1 isoforms.

### Proteic isoforms of VCAM-1 can be observed in 2D westerns of HUVECs treated with TNF or TSFZR75.30

**Figure 4:**
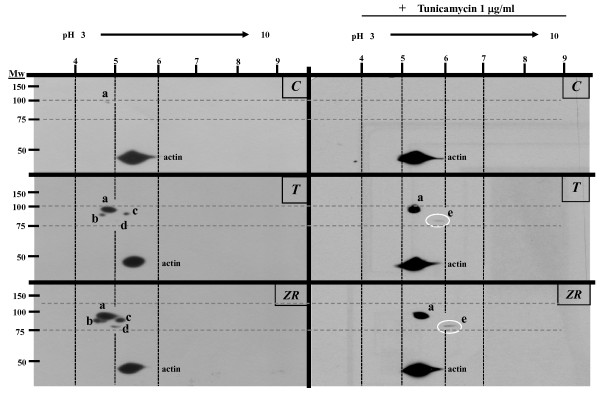
**VCAM-1 isoforms are observable in 2D western blots**. 50 μg of total protein from HUVECs was resolved in the 1^st ^dimension in a linear pH-range from 3-10 and in 10% acrylamide in the 2^nd ^dimension. *Left panels*: VCAM-1 isoforms expression in control and treated HUVEC's. *Right panels*: VCAM-1 isoforms expression observed in HUVECs with pre-treatment of tunicamycin. Control (C), TNF (T), TSFZR75.30 (ZR). The spot of actin was identified through a specific monoclonal antibody incubated at the same time as the VCAM-1 antibody. Molecular weight markers are indicated to the left of the panels and pH gradient markers are indicated above. The dotted grids were added to facilitate the comparison between images. Patterns are the representative images of two individual experiments each performed in duplicate. Numbers in parenthesis after the spot of the VCAM-1 isoform "a", reflect the relative amount with respect to samples without tunicamycin, each spot was normalized with the actin signal in the same gel, p < 0.05 (*); n = 3.

Considering that the resolution of the 2-DE gels was adequate and that the western blots analysis of VCAM-1 expression yielded intense signals, we combined these two approaches and performed 2D western blots that revealed an unexpected variety of VCAM-1 isoforms (Figure 4). We identified the only barely visible spot in the untreated control cells that displayed Mr/p*I *values close to those reported for the full length as the VCAM-1 isoform "a" (Mr ~90-95 kDa/p*I *4.8) (Figure 4C, left panels). The expression of this isoform was significantly increased when cells were treated with TNF or with TSFZR75.30. With TNF (T) two isoforms, labeled as "b" (Mr ~80 kDa/p*I *4.6) and "c" (Mr ~83 kDa/p*I *5.1-5.2), appeared below isoform "a". With TSFZR75.30 (ZR), three spots were visible: two corresponded with spots "b" and "c" and a third additional isoform labeled as "d" (Mr ~77 kDa/p*I *5.0) also became apparent.

Following the same strategy used in Figure 2 to reveal isoforms with different N-glycosylation states, we resolved using 2D westerns blots, total extracts from HUVECs pre-treated with tunicamycin. Figure 4C, right panels indicate that the faint spot we previously identified as isoform "a" in the untreated control cells disappeared after tunicamycin treatment. HUVECs treated with TNF (Figure 4T, right panels), showed a spot with the same Mr as isoform "a", but with a shift in isoelectric point from 4.8 to 5.3-5.4, along with a fifth isoform (labelled as "e") that appeared as a faint spot (Mr ~77 kDa/p*I *6.0). These isoforms (a and e) were also visible in cells treated with TSFZR75.30 (Figure 4ZR right panels). The isoform "e" was the most basic spot detected and had the same apparent Mr as isoform "d" (Table 2). Interestingly, VCAM-1 isoform "x" seen in Figure 2B displayed a similar Mr (75 kDa) as isoforms "d" and "e" (77 kDa) shown in Figure 4. Thus, in addition to the isoforms "a" and "b" previously reported in the literature, resulting from alternative splicing events, our 2D western blots analysis revealed three new isoforms defined by differential states of N-glycosylation.

**Table 2:** Molecular weights and isoelectric points of VCAM-1 isoforms.

Isoform	Mr/p*I *NCBI	Mr/p*I *treatments	Mr/p*I* tunicamycin + treatments
*a*	81 kDa/5.1	90-95 kDa/4.8	90-100 kDa/5.3-5.4
*b*	71 kDa/5.0	80 kDa/4.6	not observable
*c*	not reported	83 kDa/5.1-5.2	not observable
*d*	not reported	77 kDa/5.0	not observable
*e*	not reported	not observable	77 kDa/5.9-6.1
*actin*	42 kDa/5.3	42 kDa/5.3	42 kDa/5.3

Comparison between the values reported in the NCBI and the values observed by the treatments in the westerns of this study.

### Translocation of NF-κB to the nucleus in HUVECs treated with TSFZR75.30

**Figure 5:**
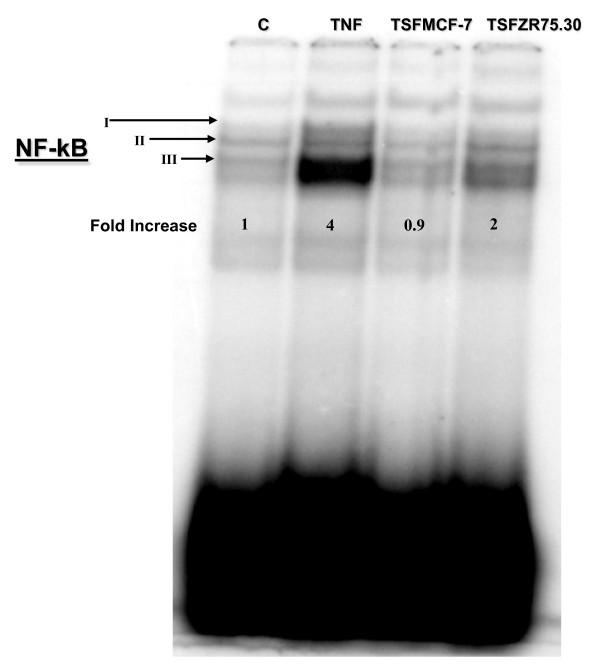
**EMSA assay of the NF-κB translocation to the nucleus in HUVECs treated with TSFZR75.30**. Nuclear extracts from HUVECs untreated and treated with TNF, TSFMCF-7 or TSFZR75.30, were subjected to EMSA using a probe for NF-κB. Roman numbers indicate NFκB/DNA complexes. Numbers below the complexes correspond to relative intensity of complex III normalized against the control signal, are the mean of two individual experiments with independent cell cultures.

Since VCAM-1 expression in response to TNF has been reported to be dependent on NF-κB activation, we tested by EMSA whether TSFZR75.30 could elicit a similar translocation of active NF-κB to the nucleus in HUVECs treated with these tumoral factors. Several DNA/NF-κB complexes were visible, but only complex III increased in cells treated with TNF or TSFZR75.30 (Figure 5). While TNF treatment led to a 4-fold increase with respect to control cells, TSFZR75.30 led to only a 2-fold increase (lane 4), despite the fact that this same treatment induces a strong pro-adhesive phenotype (Figure 1). As a negative control, we treated HUVECs with TSFMCF-7 and, as was expected, TSFMCF-7 did not promote translocation of NF-κB. In fact, a slight decrease in the signal was observed (lane 3). Hence, TSFZR75.30 was able to activate and translocate NF-κB to the nucleus.

## Discussion

The interactions of tumor cells with their neighboring endothelial cells present in their surrounding environment has emerged as an increasingly relevant factor in tumor progression during angiogenesis, intravasation at the primary tumor site, and adhesion and extravasation at the site of metastasis [26-28]. The available information indicates that the soluble factors secreted by tumor cells can alter the phenotype of different cell types, modifying their activity and provoking tissue destruction, tumoral cell migration and dissemination [29]. We have previously reported that HUVECs treated with soluble factors secreted by tumoral cells (TSF's), can adhere U937 cells and that this response is linked to the activation of NF-κB and the expression of cell adhesion molecules [19,30].

Since Jaffe established the methodology for the culture in 1973, HUVECs had been the principal model to the studies of physiological and pathological process involved endothelial cells. For instance, previous studies have investigated functional differences between HUVECs (human umbilical vein endothelial cells) and HDMECs (human dermal microvascular endothelial cells) with respect to, upregulation of adhesion molecules in response to cytokines, stimulation and expression of surface antigens or mechanical properties of leukocytes rolling. However, at the moment does not exist clear functional differences. Histological studies of the expression of adhesion molecules, as such as VCAM-1 in primary vascular tumoral tissue, can serve to compare endothelial models with the behavior of cells *in vivo *[31-33].

Our current working hypothesis is that the tumor cells can use adhesion molecules, such as VCAM-1, to interact with and adhere to the endothelial monolayers, essentially emulating leukocytes during the inflammatory reaction. In this work, we compared the increase in adhesive capacity of HUVEC's with the increased expression of different VCAM-1 isoforms.

A cell adhesion assay (Figure 1) was used to compare the pro-adhesive phenotype of HUVECs induced by TSF's. Since different tumor cell lines present variable adhesion to unstimulated endothelial cells, we used the promyelocytic human cell line U937 as a probe of the induction of pro-adhesive phenotype in response to the different TSF's. This assay showed that the factors derived from the cell line ZR75.30 (TSFZR75.30) were as effective as TNF in activating the endothelial phenotype, using the concentration of TSF's in which the percentage of adhesion (1 μg/ml-50%), was the highest and did not have differences statistically significatives, with respect to another concentrations (0.5 μg/ml-38%, 0.25 μg/ml-24%, 0.125 μg/ml-19%, 0.0625 μg/ml-18%). TSF's by other three breast cancer cell lines were prepared and tested in the same cell adhesion assay (T47D, MDA MB 435 and MDA MB 231). Addition of 1 μg/ml of TSF's from either induced different fold increase of adhesion: 1.3 ± 0, 1.6 ± 0.2, 2.2 ± 0.1 respectively.

TNF is recognized as the most important physiological stimulus for the activation of signaling pathways that lead to the translocation of NF-κB into the nucleus, for several cell types [34-36]. In HUVECs, TNF and TSFZR75.30 both induced the translocation of NF-κB to the nucleus, although the TSF's only stimulated the system by about 50% in comparison with TNF (Figure 5). However, the amount of VCAM-1 expressed was slightly higher in TSFZR75.30 treated cells, suggesting that expression of this adhesion molecule could result from the recruitment of other transcription factors activated by the mixture of elements present in the TSF's. The analysis of the mixture of TSFZR75.30 revealed very low levels of TNF, along with an abundance of cytokines such as IL-6 and IL-8 that could be responsible for NF-κB activation.

The expression of adhesion molecules, such as VCAM-1, in response to chemokines and cytokines is essential in the acute inflammatory response and represents a clear sign of an activated endothelial phenotype [35,37-39]. Unidimensional and bidimensional western blots analysis [40,41] revealed that TSFZR75.30 was able to induce the expression of VCAM-1a (Mr 81 kDa/p*I *5.1) [NCBI access number NP_001069] and VCAM-1b (Mr 71 kDa/p*I *5.0) [NCBI access number NP_542413] in HUVECs in a similar magnitude as TNF (Figure 2A).

In addition, the westerns showed four new isoforms: isoform x in uni-dimensional gels (Figure 2B) and isoforms c, d, and e in bi-dimensional gels (Figure 4). Isoforms c, d and e were present both, in cells treated with TNF as well as in those treated with TSFZR75.30, although isoform d was bearly visible in cells treated with TNF. We conclude that TSFZR75.30 promote a stronger expression of all isoforms compared to the induction mediated by TNF. In an attempt to determine if the isoforms contained N-glycosylations, we interfered with the formation of dolicholpyrophosphate *N*-acetylglucosamine, the first step in the synthesis of N-linked glycoproteins, by using tunicamycin. Under this condition, the protein portion of glycoproteins will be synthesized completely devoid of N-glycosylations [42]. VCAM-1a has six N-glycosylation sites, whereas removal of exon 5 in VCAM-1b eliminates the second of these sites. Proteins lacking N-glycosylation have been reported to have decreased stability in the endoplasmic reticulum and hence, are more easily exported and degraded in the cytoplasm by the proteasome. This is a likely explanation for the decreased cellular content of isoform "a" (40% and 70% decrease with TNF or TSFZR75.30 respectively) and the disappearance of isoform "b" in the presence of tunicamycin (Figure 2B and Figure 4).

The isoform "x" (Mr ~75-77 kDa) (Figure 2B), became visible only in the presence of tunicamycin. Considering that one N-glycosylation modification corresponds to an added weight of 3 kDa and that VCAM1a (90-95 kDa) has six N-glycosylation sites, tunicamycin treatment could lead to isoforms that are up to 18 kDa smaller. Hence, isoform "x" could correspond to the full length core protein (9 exons) lacking all N-glycosylation modifications. At this point we cannot discard that the TSFZR75.30 induce altered glycosylation compared to that indicated by TNF. The fact that tunicamycin pretreatment abolished 50% of the cell adhesion induced by TSFZR75.30 indicates that N-glycosylated proteins such as VCAM-1 play an important rol in cell adhesion, other cell adhesion molecules such as E-selectin and ICAM-1 are likely involved in this process.

We assigned VCAM-1b to the spot with a Mr ~80 kDa/p*I *4.6, the spot with Mr ~83 kDa/p*I *5.1-5.2 we labeled as isoform "c", which could correspond to the core protein of isoform "b", with different pI resulting from differential states of sialo-glycosylation at any of the five remaining N-glycosylation sites. Alternatively, isoform "c" could also result from the loss of exons 2 or 8, leading to a protein with a similar Mr as "b", but with an increase in the density of charge due to the preservation of all the reported glycosylations sites (Figure 2A).

The appearance of isoform "d", which was overexpressed in cells treated with TSFZR75.30, could be of potential clinical use as a biological marker for indicating the abnormal activation of endothelial cells by tumoral factors. Isoform "d" had the lowest molecular weight, which was suggestive of a smaller protein generated by alternative splicing or proteolytic processing. Interestingly when we interfered the process of N-glycosylation, isoform "d" disappeared, and a new isoform "e" (Mr ~77 kDa/p*I *6.0) appeared. The fact that these two isoforms ("d" and "e") have the same apparent Mr suggests that they both correspond to the same core protein. It is likely, that the isoform "x", identified in the Figure 2-B, corresponds to isoform e, since both were visible only in the presence of tunicamycin. According to the reported exon structure of VCAM-1, we evaluated the possibilities for the expected proteins when exons 2 (92 amino acids), 3 (107 amino acids), or 8 (89 amino acids) were eliminated. The predicted proteins had the following Mr/p*I *values: 68928 kDa/5.13, 66997 kDa/5.14, and 69376 kDa/5.01, indicating that none of them could produce the observed "e" isoform, which had a Mr/p*I *value of ~77 kDa/6.0. This analysis further supports the idea that isoform "e", is encoded by all nine exons but lacks all N-glycosylation modifications. In addition, although we cannot discard other types of posttranslational modifications such as phosphorylation that could also explain the differences in pI of the different isoforms, these modifications have not been previously described for VCAM-1. Expression of VCAM-1 isoforms in tumors has not been well studied. In the past half-century, numerous studies have dealt with the effects of TSF's on endothelial cells. These studies have demonstrated that malignant cells produce a host of factors, most notably VEGF, that favor growth and vascular permeability, facilitating the spread of tumors [43-45]. In addition to cytokines and chemokines, our study also detected significant amounts of VEGF secreted by the breast cancer cell line ZR75.30. The complex mixture of soluble factors secreted by these cells reflects the multifactorial nature of signals emitted by tumor cells that can influence endothelial behavior [46]. The specific combination of cytokines, chemokines and growth factors, observed in the TSF's could serve as a signature to distinguish between tumor cells with different metastatic or invasive potentials in breast cancer.

## Conclusion

Although it has been known for some time that altered glycosylation patterns of cell surface proteins, in particular increased sialylation, are associated with cancer cell adhesion, mobility, and invasion, only recently the functional significance of these changes has begun to be understood. This study documents variants of the N-glycosylation state of VCAM-1 that can be induced in normal endothelial cells exposed to tumoral soluble factors derived from human breast cancer cells that could contribute to cell-cell adhesion and hence to malignancy [47-49].

## Methods

### Cell cultures

Human umbilical vein endothelial cells (HUVECs) were isolated and cultured [50,51] by mixing cells from two or three human umbilical cords. The protocol to obtain the cells was approved by the ethics committee of the Gynecology and Obstetrics Hospital number 4 "Luis Castelazo Ayala", Mexican Institute of Social Security (IMSS), follow the principles of the Helsinki Declaration for human experimental research. Informed consent was also obtained. The culture medium was M-199 (Gibco BRL, Grand Island, NY), supplemented with 10% fetal bovine serum (In vitro, D.F. Mexico), 1% glutamine (SIGMA, St Louis, MO), 20 μg/ml endothelial mitogen (Biomedical technologies, Stoughton, Ma), 100 μg/ml heparin (SIGMA, St Louis, Mo), and 100 U/ml penicillin/streptomycin (Gibco BRL, Grand Island, NY). Cells were grown on plastic tissue culture plates (Costar, Cambridge, MA) under an atmosphere of 95% humidity and 5% CO_2 _at 37°C. The cell culture reached confluence approximately 1 week after plating and presented a characteristic cobblestone appearance; cell cultures were used for all the reported experiments within their first passage. The myeloma cell line (U937) and breast cancer cell lines (ZR 75.30 and MCF-7) were obtained from ATCC, cultured in RPMI media supplemented with 10% FBS, and grown under endotoxin-free conditions.

### Production of tumoral soluble factors

Breast cancer cell lines were cultured until they reached 100% confluence. The cell layer was first washed 10 times with phosphate-buffered saline (PBS) and DMEM (1:1 v/v) in order to remove serum components. Then, the flasks were incubated with 20 ml of serum free RPMI. After 48 h, the culture medium (containing the soluble products derived from the breast cancer cell lines) was collected and lyophilized following centrifugation. The resulting powder was dissolved in 1/10 of the original volume and dialyzed using a PM-10 ultra-filtration membrane (Millipore, Bedford, MA). The protein concentration was determined using the commercial Bradford reagent assay (Bio-Rad, Hercules, CA). The resulting concentrated preparation containing the tumoral soluble factors from the breast cancer cell line ZR75.30 (TSFZR75.30) or the breast cancer cell line MCF-7 (TSFMCF-7), was kept at 4°C until further use.

### Adhesion assay

A suspension of U937 cells (1 × 10^6 ^cells/ml) was radio labeled with thymidine (^3^H) (1 μCi/ml) (NEN, Boston, MA) for 48 h. Aliquots of the labeled cell suspension (250 × 10^3 ^cells/250 μl) were added to previously prepared wells containing HUVECs that had been grown and stimulated for 3 h. The assay was performed in 48 well plates. After an additional 3 h of co-incubation, all the non-adherent U937 cells were removed by aspiration, followed by two washes with PBS. The adherent cells were then immediately lysed with 500 μl of 0.2 M NaOH and the radioactivity was measured in a scintillation counter (Beckman LS6000SC, St Louis, MO) [30].

### Tunicamycin treatment

The tunicamycin concentration used was based on its N-glycan inhibitory effects on human cell cultures. HUVECs monolayers in Petri dishes were incubated with 1 μg/ml tunicamycin (SIGMA, St Louis, MO) for 3 h followed by TNF or TSFZR75.30 treatment. The cells were harvested 6 h later [52].

### Western blots

Total protein concentration was determined using the commercial Bradford reagent assay (Bio-Rad, Hercules, CA). 20 μg of total protein was used for the detection of VCAM-1. Samples were first boiled in sample buffer (125 mM Tris-HCl pH 6.8, 1% v/w SDS, 10%v/v glycerol, 0.1% bromophenol blue, 2% v/v 2beta-mercaptoethanol) for 5 min and separated by 10% SDS-PAGE. Then, the gels were transferred to PVDF membranes (Bio-Rad, Hercules, CA) using a Trans-Blot Cell system (Bio-Rad, Hercules, CA) in transfer buffer (25 mM Tris, 190 mM glycine, 10% methanol) at 40 V overnight [53]. The following day, the membranes were probed for 1 h with mouse anti-human VCAM-1 (CD106) antibody (sc 13160 Sta. Cruz, Sta. Cruz, CA) diluted 1:500 in TBS buffer (150 mM NaCl, 20 mM Tris, 0.1% Tween, 1% BSA, pH 7.5). After washing, the membranes were incubated for 1 h with horseradish peroxidase linked to antimouse immunoglobulin (Pierce Rockford, IL). The signals were detected by enhanced chemiluminescence using the supersignal system (Pierce Rockford, IL) and quantified by densitometry. As a control, actin was simultaneously detected, using a mouse anti-human actin antibody. The antibody was diluted 1:1000 and developed using the same secondary antibody and chemiluminescence system previously described [54].

### Two-dimensional gel electrophoresis

Confluent cells were either left untreated or treated for 6 h with TNF or TSFZR75.30. Cells were rinsed twice with PBS containing Ca^++ ^and Mg^++^, and harvested in lysis buffer containing 7 M urea, 2 M thiourea (SIGMA, St Louis, MO), and 4% w/v CHAPS (Bio-Rad, Hercules, CA). Samples were centrifuged at 18000 g for 5 min, the supernatants were recovered, and the pellets were discarded. The supernatants were then transferred to a new eppendorf tube, and the salts were removed from the samples using a 2-D Clean-up Kit (Amersham Biosciences, San Francisco, CA). 50 μg of total protein was mixed with DeStreak rehydration buffer and 0.5% IPG buffer, pH 3-10 (Amersham Biosciences Uppsala, Sweden) and applied to 7 cm IPG strips, pH 3-10 (Amersham Biosciences, San Francisco, CA), which were allowed to rehydrate for 15 h at room temperature. Separation on the first dimension was carried out using an IPGphor II isoelectric focusing system (Amersham Biosciences, Uppsala Sweden), as described by Görg in 1988 [17,55]. After the first dimension, the strips were balanced in two steps: (i) 15 min in a solution containing 6 M urea, 50 mM Tris (pH 8.8), 30% glycerol, 2% SDS, and 70 mM DTT, and (ii) 15 min in a similar solution contain 140 mM iodoacetamide. After mounting the strips on 10% acrylamide gels, vertical electrophoresis was carried out using the Miniprotean III Bio-Rad system (Bio-Rad Hercules, CA). Proteins in the gels were visualized with the Deep-Purple stain solution (Amersham Biosciences, Bucks, UK) [1].

### Deep Purple gels stain

Gels were stained according to instructions of the supplier http://www.amershambiosciences.com/. Briefly, the gels were first washed with distilled H_2_O for 30 min, and then fixed in a solution of 7.5% acetic acid and 10% ethanol overnight. The next day, gels were stained with 5 ml of deep purple diluted in 200 mM Na_2_CO_3 _for 30 min and then rinsed twice, 20 min each, with 50 ml distilled H_2_O containing 7.5% acetic acid. After that, gels were briefly washed in new last solution and imaged immediately in a Typhoon 9410 high performance analyzer™ using the 532 nm excitation laser. Protein spots were detected and quantified as fluorescent volumes; such a volume is the sum of the intensity of all pixels within the defined spot area. The gels with the highest number of spots were selected as reference gels and a combined warping with matching algorithm was used to create an average gel [56,57].

### 2D Western blots

50 μg of total protein was used for the detection of VCAM-1/actin for each of the treatments. The samples were separated in first and second dimension following the previously described protocols and transferred to an Immuno-blot PVDF membrane (Bio-Rad, Hercules, CA). Membranes were blocked with 0.5% milk for 1 h and probed with mouse antihuman CD106 (VCAM-1) antibody (sc 13160 Sta. Cruz, Sta. Cruz, CA) diluted 1:500 in TBS with 3% BSA overnight at 4°C, following previously described western blots protocols.

### Electrophoretic mobility shift assay (EMSA)

Nuclear protein extracts were obtained after treatments, in which the cells were washed, scraped, and pelleted at 4°C and then frozen in ethanol-dry ice for 1 min. The cells were immediately resuspended in 100 μl of buffer A (10 mM HEPES, 10 mM KCl, 1.5 mM MgCl2, 1 mM DTT, pH 7.9) and incubated for 10 min at 4°C. Nuclei were microcentrifuged, resuspended in 30 μl of buffer B (20 mM HEPES, 400 mM NaCl, 1.5 mM MgCl2, 0.2 mM EDTA, 25% glycerol, 1 mM DTT, 0.5 mM PMSF pH 7.9), and incubated on ice for 30 min. Following another 20 min microcentrifuge step, the supernatant (nuclear protein extract) was diluted with 30 μl of HDKE buffer (20 mM HEPES, 50 mM KCl, 25% glycerol, 0.2 mM EDTA, 1 mM DTT, 0.5 mM PMSF, pH 7.9). 10 μg of the nuclear protein extracts were incubated with γ-ATP (32P) labeled oligonucleotide containing the decameric κB site (5'AGTTGAGGGGACTTTCCCAGGC 3') (Santa Cruz, Sta. Cruz, CA). The binding reactions were carried out by incubating on ice for 40 min in reaction buffer (20 mM HEPES, 50 mM KCl, 20% glycerol, 0.2 mM EDTA, 0.5 mM PMSF, 1 mM DTT, 1 μg/μl BSA, 1 μg/μl poly-dI-dC) (Amersham Biosciences, Uppsala Sweden). The reaction mixture was loaded onto a 7.5% non-denaturing polyacrylamide gel, and was resolved at 120 V for 4 h. The gel was dried and the DNA protein complexes were visualized by exposing the gel to a storage phosphor screen, imaged on a Storm Phosphorimager (Molecular Dynamics, San Francisco, CA), and analyzed with the ImageQuant software (Molecular Dynamics, San Francisco, CA) [58].

### Bio-Plex assay

The Bio-Plex suspension array system (Bio-Rad, Hercules, CA) is a microsphere-based immunoassay, which utilizes Luminex™ beads coupled to specific antibodies, as an analyte capture platform [59]. In total, 50 μl of tumoral soluble factors secreted by the breast cancer cell line ZR75.30, was used for detecting the contents of secreted factors. The samples were added in duplicate to 96-well plates containing polystyrene beads from the 27-plex assay kit, and the beads were filter-washed twice with Bio-Plex wash buffer using a vacuum manifold (Millipore, Bedford, MA). Human cytokine standards were prepared in a range of concentrations from 32,000-0.2 pg/ml, added to the antibody-conjugated beads, and incubated in the dark on a platform shaker for 30 min. Following incubation, the samples and standards were removed by vacuum, and the beads were filter-washed three times with Bio-Plex wash buffer. Afterwards, a 1:50 dilution of biotinylated detection antibody was added to the beads, followed by incubation in the dark on a platform shaker for 30 min. The beads were washed three times and reacted with a 1:100 dilution of streptavidin-phycoerythrin (PE) for 10 min. The beads were washed three times as described above, re-suspended in Bio-Plex assay buffer, and analyzed on a Bio-Plex plate reader [59,60].

### Bioinformatic tools

Sequences of VCAM-1 were taken from the National Center for Biotechnology Information (NCBI) with the identification numbers: NP_001069 (VCAM-1a) and NP_542413 (VCAM-1b). Determinations of Mr/p*I *during the analysis of the VCAM-1 isoforms were done based on the same sequences using the Expert Protein Analysis System (ExPASy) proteomics server of the Swiss Institute of Bioinformatics (SIB).

### Statistical analysis

All data sets were analyzed using two-tails Student's *t *test.

## Abbreviations

(HUVECs): Human umbilical vein endothelial cells; (TSF's): Tumoral soluble factors; (TNF): Tumoral necrosis factor; (VCAM-1): Vascular cellular adhesion molecule 1; (2D PAGE): Two-dimensional polyacrylamide gel electrophoresis; (Mr): Relative mass; (p*I*): Isoelectric point.

## Authors' contributions

*DMS*: carried out all the experiments, their analysis and wrote the manuscript. *JLV*: designed, carried out experiments and provided scientific advice to the manuscript. *SF*: isolated and established HUVECs cultures. *IM*: analyzed 2DE patterns with the software Image Master 2D Platinum. *LM*: performed the EMSA assays. *AEN *and *FVO*: provided and performed the Bio-Plex assays for the TSF's. *AZD*: designed and directed the phases of the research, analyzed the data, and critically reviewed and wrote the manuscript. All authors read and approved the final manuscript.
